# Circ_0089761 accelerates colorectal cancer metastasis and immune escape via miR‐27b‐3p/PD‐L1 axis

**DOI:** 10.14814/phy2.70137

**Published:** 2024-12-04

**Authors:** Qizhong Gao, Xiaowei Cheng, Xiang Gao

**Affiliations:** ^1^ Department of Gastrointestinal Surgery Affiliated Hospital of Jiangnan University Wuxi Jiangsu China; ^2^ Internal Medicine Oncology Affiliated Hospital of Jiangnan University Wuxi Jiangsu China

**Keywords:** circ_0089761, colorectal cancer, miR‐27b‐3p, PD‐L1

## Abstract

Circular RNAs have been implicated as critical regulators in the initiation and progression of colorectal cancer (CRC). This study was intended to elucidate the functional significance of the circ_0089761/miR‐27b‐3p/programmed cell death ligand 1 (PD‐L1) axis in CRC. Our findings indicated that circ_0089761 expression was significantly elevated in CRC tissues and cell lines. Furthermore, the high expression of circ_0089761 was correlated with TNM stage and tumor size. Silencing circ_0089761 inhibited CRC cell proliferation, migration, and invasion, and increased apoptosis. Mechanistically, circ_0089761 facilitated its biological function by binding to miR‐27b‐3p to upregulate PD‐L1 expression in CRC. Coculture experiments confirmed that low expression of circ_0089761 impeded CD8 + T cell apoptosis and depletion, activated CD8 + T cell function, and increased secretion of the immune effector cytokines IFN‐γ, TNF‐α, perforin, and granzyme‐B. MiR‐27b‐3p inhibition or PD‐L1 overexpression partially impeded CD8 + T cell function. The circ_0089761/miR‐27b‐3p/PD‐L1 axis is postulated to exert pivotal functions in the mechanistic progression of CRC. Furthermore, it holds promising prospects as a feasible biomarker and therapeutic target for CRC.

## 1 INTRODUCTION

Colorectal cancer (CRC) is a malignant tumor that emerges from colon or rectum and represents one of the leading causes of cancer‐related deaths, posing a significant global health concern (Li et al., 2021). Timely diagnosis plays a crucial role in CRC successful treatment, for early‐stage cases could be cured (Siegel et al., 2017). Unfortunately, patients often do not exhibit noticeable symptoms until the disease has advanced, which greatly impacts the effectiveness of the clinical interventions (Biller & Schrag, 2021). Moreover, the development of chemotherapy and targeted therapy resistance poses a significant challenge in managing advanced colorectal cancer (Weng et al., 2022). Certain risk factors for CRC have been identified, including age (Patel et al., 2022), family history (Kastrinos et al., 2020), smoking (Bai et al., 2022), obesity (Bardou et al., 2013), and physical inactivity (Keum & Giovannucci, 2019). However, the exact pathogenesis of CRC remains unknown.

It has been confirmed that noncoding RNAs (ncRNAs) exhibit critical roles in diverse biological processes (Wang et al., 2019). Extensive research over the past decade has illuminated the diverse functions of ncRNAs and their involvement in various cancers, including bladder cancer (Sanya & Onésime, 2023), thyroid cancer (Ghafouri‐Fard et al., 2020), and CRC (Lulli et al., 2022). Circular RNAs (circRNAs) have been validated as promising biomarkers for cancer diagnosis due to their abundance, stability and stage‐specific expression pattern during development (Conn et al., 2024). For example, circNRIP1 acted as the biomarker of gastric cancer (Zhang et al., 2019); circ_0003258 was confirmed to be a biomarker in prostate cancer (Yu et al., 2022); circUSP7 had the function of biomarker in lung cancer (Chen et al., 2021). Among the various ncRNAs, microRNAs (miRNAs), and circRNAs have emerged as key players in cancer biology (Sakshi et al., 2021). Previous research has constructed circRNA‐miRNA‐mRNA networks in cancers (Heidari‐Ezzati et al., 2024; Hossain et al., 2022; Zhang, Bai, et al., 2021), elucidating their roles in tumor prognosis and pathogenesis. It is proposed that circRNAs bind to miRNAs, thereby regulating each other's expression and affecting tumor development and progression (Gao et al., 2022). For example, circSHKBP1 regulates the VEGF via miR‐582‐3p in gastric cancer (Xie et al., 2020). Overall, circRNA‐miRNA‐mRNA axis has crucial roles in cancer progression. Their complex interactions and functions in cancer biology will provide valuable insights into novel therapeutic targets and diagnostic biomarkers.

Programmed cell death ligand 1 (PD‐L1), a cell surface protein, plays crucial roles in the regulation of immune responses through interaction with programmed cell death protein 1 (PD‐1) (Topalian et al., 2020). Cancer cell‐derived PD‐L1 regulates CD8 + T cell activity, and mechanistically, high levels of PD‐L1 in cancer cells induce CD8 + T cell dysfunction, leading to immune evasion and failure of immune surveillance (Fang et al., 2021). PD‐L1 has association with prognosis and resistance across multiple cancer types, including lung cancer (Patil et al., 2022), melanoma cancer (Serratì et al., 2022), bladder cancer (Jing et al., 2022), and breast cancer (Vranic et al., 2021). PD‐L1 enrichment accelerates cancer cell growth and motility and maintains stemness in breast cancer cells to promote drug resistance (Vranic et al., 2021). Previous studies have confirmed that their targeted inhibitors have potential in the treatment of certain malignancies (Yi et al., 2022). Moreover, additional functions of PD‐L1 in tumorigenesis have also been elucidated (Huang et al., 2022), cancer progression (Liu, Reck, et al., 2021; Xie et al., 2019), proliferation (Shen et al., 2022), angiogenesis (Xie et al., 2019), invasion (Shen et al., 2019), metastasis (Loibl et al., 2021), tumor cell survival (Reck et al., 2021), chemotherapy resistance (Jiang et al., 2021), and immune evasion (Tauriello et al., 2018). This pathway holds potential as feasible biological markers and therapeutic targets for CRC. In sum, the role of PD‐L1 is imperative for improving personalized treatment approaches and identifying novel therapeutic targets to enhance patient outcomes in cancer treatment.

Circ_0089761 is a novel circRNA that exhibits aberrant expression in several human diseases, including esophageal cancer. However, circ_0089761 has not been associated with colorectal cancer. Therefore, we conducted the following study to provide a basis for the diagnosis and treatment of CRC. Here, we found that circ_0089761 competed with the key immunomodulatory gene PD‐L1 for miR‐27b‐3p and deregulated the inhibitory effect of miR‐27b‐3p on PD‐L1, which led to an increase in PD‐L1 expression and enhanced metastatic ability of CRC cells. Thus, the circ_0089761/miR‐27b‐3p/PD‐L1 axis may provide a new therapeutic target for the treatment of CRC.

## 2 METHODS

### 2.1 Tissues

Cancerous and adjacent noncancerous tissues of 75 CRC patients were collected at our hospital, which were histologically confirmed. Written informed consent was obtained from all participants prior to enrollment. They were clinically divided into stage I–IV based on AJCC staging criteria: stage I + II (*n* = 13), stage III (*n* = 25), and stage IV (*n* = 37). The tissues were kept at −80°C. This study adhered to the principles outlined in the *Declaration of Helsinki* and received approval from the Ethics Committee of Affiliated Hospital of Jiangnan University (LS2022067).

### 2.2 Cells and cell transfection

The CRC (HCT116 and SW480) cells, vectors and inhibitors were procured from Pricella (Wuhan, CL‐0096, CL‐0223). Dulbecco's modified Eagle's medium (Invitrogen, 11965092, USA) was utilized to culture cells (10% fetal bovine serum, 1% penicillin and streptomycin), which were kept at 37°C in a 5% CO_2_ incubator.

According to manufacturer's protocol, si‐circ_0089761 (Ribobio, P202305210009, China), si‐negative control (si‐NC), miR‐27b‐3p mimic, NC mimic, pcDNA3.1‐circ_0089761, pcDNA3.1‐NC, and pc‐DNA3.1‐PD‐L1were transfected into cells with lipofectamine 2000 (Invitrogen, 11668, USA). Lentiviral vectors (PLKO.1, QCP0258, Qcheng bio, China) containing circ_0089761‐specific short hairpin RNA (sh‐circ_0089761) or the corresponding control (sh‐NC) were synthesized by Ribobio (PA20230424006) and utilized for the stable downregulation of circ_0089761 in CRC cells. Subsequently, puromycin was used to screen transfected cells. RT‐qPCR was utilized for assessment of transfection efficiency.

### 2.3 RT‐qPCR

Trizol reagent (Thermo Fisher, 15596018CN, USA) was used to isolate total RNA. High‐Capacity cDNA Reverse Transcription Kit (Thermo Fisher, 4368814, USA) was utilized to reverse transcription. SYBR Green Kit (Thermo Fisher, 4309155, USA) was used to measure the genes expression. The gene expression was evaluated using 2^−ΔΔct^ method. GAPDH and U6 were used as internal reference genes for mRNA/circRNA and miRNA. The primers were provided in Table 1.

**TABLE 1:** Primer sequences of RT‐qPCR assays.

Target gene	Primer sequences (5′‐3′)	
	Forward	Reverse
circ_0089761	GCGCTGCATGTGCCATAGGC	GGAGGACAACCAGTAAGCTACC
GAPDH	AAGGTCGGAGTCAACGGATTTG	CCATGGGTGGAATCATATTGGAA
U6	CACTGTTCCACCCCTCAGAGC	GCCACTTGTCGGCGATAAGG
miR‐27b‐3p	TTATGCCCAGCGATGACC	GGCTCCAACTTAACTGTCCC
PD‐L1	TGCCGACTACAAGCGAATTACTG	CTGCTTGTCCAGATGACTTCGG

### 2.4 Immunohistochemistry (IHC)

Immunohistochemistry Kit (Sangon Biotech, D601037, China) was utilized to stain CRC tissue. CRC tissue was fixed in ethanol and subsequently embedded in paraffin for sectioning. To deparaffinize the tissue sections, xylene was used, followed by dehydration in a gradient of alcohol. 3% hydrogen peroxide was included to inhibit the activity of endogenous peroxidase. Afterward, the tissue sections underwent antigen retrieval through microwave treatment in citrate buffer. The sections were incubated with an antihuman PD‐L1 antibody (Beyotime, AG8621, 1:100, Shanghai) for 24 h. Subsequently, the tumor sections were subjected to incubation at 37°C for 15 min with a horseradish peroxidase‐labeled secondary antibody (Beyotime, A0208, 1:50, Shanghai). DAB staining kit (Beyotime, P0203, Shanghai) was utilized to stain the sections. The staining results were snapped under microscope (Leica Microsystems, ×100, Germany).

### 2.5 Cell counting kit‐8 (CCK‐8)

CCK‐8 kit (Sigma, 3423972, China) was utilized to measure the cell proliferation. 2 × 10^3^ tranfected CRC cells were counted and then suspended in medium. The suspension was incubated with CCK‐8 reagent (10 μL) at 37°C for 4 h. The absorbance was measured to determine the cell proliferation at 450 nm by a microplate reader (Thermo Scientific, Multiskan MK3).

### 2.6 Transwell assay

The cell invasion assay was conducted using a Transwell chamber (8 μm, Corning, USA) coated with Matrigel (Biosharp, BL1834C, China). 5 × 10^4^ tranfected cells were seeded in the upper chamber with serum‐free medium. The lower chamber was filled with medium containing 10% FBS (Pricella, 164210, Wuhan). After 48 h of incubation, noninvading cells were removed using PBS, and the invading cells were fixed with formaldehyde and stained with 0.5% crystal violet (Beyotime, C0121, Shanghai) at room temperature for 30 min. A transwell chamber without Matrigel was used and other steps were same in cell migration assays. Finally, images were taken, and cell counts were performed using an inverted microscope system (Olympus, CKX53, CellSens Imaging Software).

### 2.7 Apoptosis assay

Flow cytometry and Annexin V‐PE apoptosis kit (Biovision, K203‐25, China) was utilized to investigate cell apoptosis. Initially, the cells (1 × 10^6^) underwent trypsin digestion, followed by washing with PBS and subsequent resuspension in binding buffer. Then, samples were supplemented with 5 μL Annexin V‐FITC and 10 μL PE for 15 min. BD Accuri™ C6 Plus flow cytometer (BD Biosciences) was used to analyze cell apoptosis.

### 2.8 Bioinformatics analysis and dual‐luciferase reporter assay

Starbase was employed to predict the binding sequence in circ_0089761/miR‐27b‐3p/PD‐L1 axis. The luciferase assay kit (Biovision, K801‐200, China) was utilized to examine the luciferase activity. The wild‐type and mutant sequences of circ_0089761 were inserted into the pGL3 control vector (Youbio, VT1555, China) to construct the corresponding luciferase reporter gene vectors. SW480 cells were divided into several groups, including a negative control group, a miR‐27b‐3p mimic group, and a miR‐27b‐3p mimic+circ_0089761‐wt or mut group.

The wild‐type and mutant sequences of PD‐L1 were inserted into the pGL3 control vector to construct the corresponding luciferase reporter gene vectors. Cells were divided into several groups, including a negative control group, a miR‐27b‐3p mimic group, and a miR‐27b‐3p mimic+PD‐L1‐wt or mut group.

The miR‐27b‐3p mimic and respective luciferase reporter gene vectors were transfected using Lipofectamine2000 transfection reagent following the manufacturer's instructions. The luciferase activity was quantified by the dual luciferase Reporter gene assay system after 48 h. Subsequently, luciferase values were normalized against the internal control sea kidney luciferase values, and the fold change was calculated accordingly.

### 2.9 Western blotting

RIPA lysis buffer (MCE, HY‐K1001, China) was used to isolate total protein. The protein concentration was measured using BCA protein assay kit (GLPBio, GK10009, USA). Then, 10% SDS‐PAGE was utilized for proteins separation. The proteins were transferred to PVDF membranes (Millipore, IPVH00010, USA). The bands were blocked in 5% skim milk at 37°C for 1 h. After that, the membranes were subjected to overnight incubation with the addition of primary antibodies at 4°C. The primary antibodies: anti‐PD‐L1 (ab205921, 1:20000, Abcam), anti‐E‐cadherin (ab227639, 1:25, Abcam), anti‐N‐cadherin (ab76011, 1:1000, Abcam), anti‐vimentin (ab92547, 1:5000, Abcam), and GAPDH (ab181602, 1:10000, Abcam). Subsequently, the membranes interacted with HRP‐coupled goat secondary antibody (ab6721, 1:20000, Abcam) for 2.5 h. After the addition of the luminescent solution, Enhanced chemiluminescence Western blotting detection reagents (Thermo Fisher, 32209, USA) was utilized to visualize the target protein bands.

### 2.10 CD8+ T cell isolation and coculture

Human CD8 + T cells were purified from peripheral blood mononuclear cells of healthy donors using the CD8 + T cell sorting kit (Thermo Fisher, 8804‐6812‐74, USA). Isolated CD8 + T cells were planted into the Transwell upper chamber at a ratio of 1 × 10^6^ cells/well, and then the Transwell upper chamber and CD8 + T were transferred into a 6‐well plate containing 2 × 10^5^ cells/well of CRC cells, and coculture was continued for 72 h.

### 2.11 CD8 + T cell proliferation and apoptosis assay

The proliferative capacity of CD8 + T cells was assessed by flow cytometry. To analyze the expression of Ki67, CD8+ T cells were fixed with 4% paraformaldehyde for 30 min and permeabilized with 0.1% Triton X‐100 for 2 min, then washed and resuspended in PBS buffer. Anti‐Ki67 antibody (Abcam, ab197234, 1:50) was added to the cell suspension and maintained in the dark for 30 min. After washing, the rate of Ki67 positive cells was detected by flow cytometry.

The level of apoptosis in CD8 + T cells was assessed by TUNEL staining. The suspension of CD8+ T cells was incubated with TUNEL reaction reagent (Roche, 11684817910, USA) for 1 h. After washing, the stained cells were detected by flow cytometry.

### 2.12 Enzyme‐linked immunosorbent assay (ELISA)

Supernatants of CD8 + T cells were collected after 48 h of culture with CRC cells. IFN‐γ (Beyotime, PI521, China), TNF‐α (Beyotime, PT518, China), Perforin (Abcam, ab46068, USA), and Granzyme‐B (Merck, RAB1498, USA) in CD8 + T cell culture supernatants were determined by ELISA kit according to the instructions. Optical density was measured at 450 nm using an automated microplate reader.

### 2.13 Mice model

Five‐week‐old female mice (C57BL/6) were obtained from Sangon Biotech (Shanghai, China) and raised under pathogen‐free conditions. Stable knockdown of circ_0089761 cells (sh‐circ_0089761, CT26 cells, 1 × 10^7^, 0.1 mL) were subcutaneously injected into the left back region of mice to establish mouse models with tumors under the skin. Mice were categorized into two groups (sh‐NC and sh‐circ_0089761 groups, *n* = 5). Tumor size was assessed weekly (volume = (length×width^2^)/2). 5 weeks later, all mice were killed by cervical dislocation. Tumor tissue samples were dissected, weighed, and collected for further study.

### 2.14 Statistical analyses

All data were analyzed and processed with GraphPad Prism 9.0. Means ± standard deviation (SD) of results were obtained from three independent experiments. Pearson's correlation assay was utilized to examine the correlation among circ_0089761, miR‐27b‐3p, and PD‐L1 in CRC. The statistical significance of the differences was evaluated by a Student's *t*‐test or a two‐way analysis of variance (ANOVA) as indicated in the corresponding figure legends. *p* < 0.05 indicated statistical significance.

## 3 RESULTS

### 3.1 Expression of circ_0089761, miR‐27b‐3p, and PD‐L1 in CRC tissues

Previous studies have demonstrated the identification of certain clinical characteristics in CRC, including age, sex, tumor size, and TNM staging. To investigate their correlations, we analyzed the circ_0089761 expression in a cohort of 75 patients. Our findings indicated a significant association between circ_0089761 and tumor size (*p* = 0.0339) as well as TNM staging (*p* = 0.0051) (Table 2). However, no significant association was noted between circ_0089761 and other clinical characteristics, including age, male, and tumor site.

**TABLE 2:** Correlations between circ_0089761 and clinical characteristics of CRC patients.

Clinicopathologic characteristics	*n*	circ_0089761		
		Low (*n* = 37)	High (*n* = 38)	*p* **Value**
Age (years)				
< 60	30	14	16	0.8147
≥ 60	45	23	22	
Sex				
Male	39	21	18	0.4910
Female	36	16	20	
Tumor size				
<30 mm	29	19	10	0.0339
≥30 mm	46	18	28	
Tumor site				
Colon	40	21	19	0.6457
Rectum	35	16	19	
TNM staging				
I + II	13	11	2	0.0051
III + IV	62	26	36	

**FIGURE 1:**
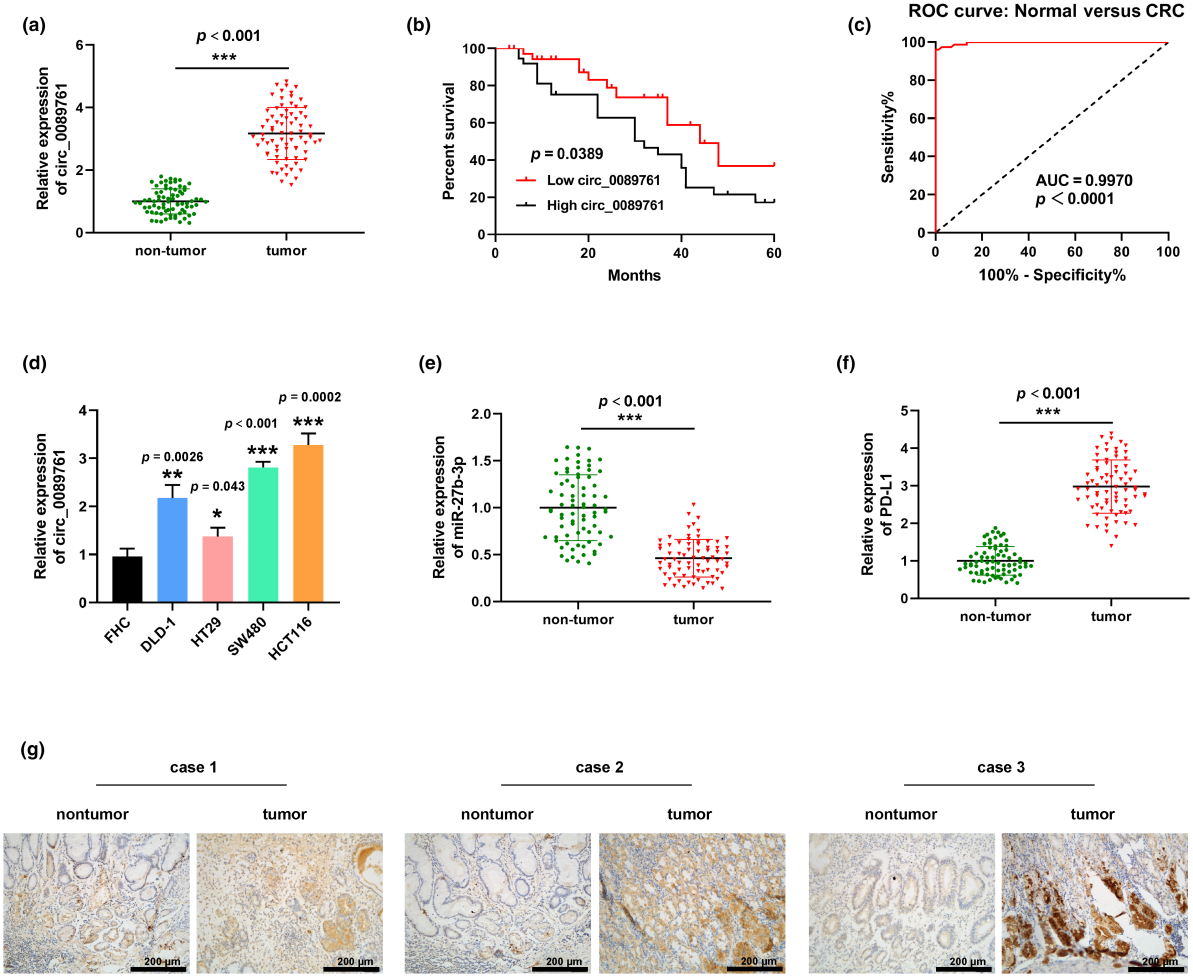
Circ_0089761, miR‐27b‐3p, and PD‐L1 expression in CRC. (a) RT‐qPCR was used to examined circ_0089761 in 75‐paired CRC tumor and adjacent nontumor sample. (b) Relationship between circ_0089761 expression level and overall survival in colorectal cancer patients. (c) ROC curves based on circ_0089761 levels. (d) Analysis of circ_0089761 expression in DLD‐1, HT29, SW480, and HCT116 cells. (e) RT‐qPCR was utilized to examined miR‐27b‐3p in 75‐paired CRC and adjacent nontumor sample. (f) RT‐qPCR was utilized to examined PD‐L1 in 75‐paired CRC tumor and adjacent nontumor sample. (g) The immunohistochemistry staining was conducted to measure the PD‐L1 protein from three CRC patients. Scale bars: 200 μm. *p < 0.05 versus nontumor or FHC; **p < 0.01 versus FHC; ***p < 0.001 versus FHC.

Abnormal expression of genes was relative with carcinogenesis and tumor formation. First, bioinformatics tools (Starbase, Targetscan, miRDB, circbase) were used to predict potential downstream targets of circ_0089761, and the results showed that circ_0089761 targets the miR‐27b‐3p/PD‐L1 axis. To investigate the participation of circ_0089761/miR‐27b‐3p/PD‐L1 axis in CRC, a series of experiments were conducted using RT‐qPCR and IHC techniques. Firstly, the expression of circ_0089761, miR‐27b‐3p, and PD‐L1 was evaluated in CRC and nontumor tissue samples through RT‐qPCR analysis. Our findings revealed that compared with the nontumor tissues, circ_008976 (Figure 1a) and PD‐L1 (Figure 1f) expression of tumor were significantly increased. Vise versa, miR‐27b‐3p in CRC tumor samples was significantly decreased (Figure 1e). Kaplan–Meier analysis of the data showed that CRC patients with high circ_0089761 levels had significantly lower overall survival than those with low circ_0089761 levels (Figure 1b). As shown in the ROC curve in Figure 1c, circ_0089761 could be used to differentiate CRC patients from cancer‐free control individuals, with an AUC of 0.997. In addition, CRC cell lines (DLD‐1, HT29, SW480, and HCT116) presented a higher expression of circ_0089761 than normal colonic epithelial cells (FHC) (Figure 1d). IHC analysis was applied to assess the expression of PD‐L1 protein in CRC and nontumor tissues. Notably, a substantial upregulation of PD‐L1 protein was noted in CRC tumors in comparison to nontumor tissues (Figure 1g). All results provided valuable perspectives on the potential role of the circ_0089761/miR‐27b‐3p/PD‐L1 axis in CRC, focusing on the changes in expression levels and protein localization.

### 3.2 The expression of circ_0089761, miR‐27b‐3p, and PD‐L1 was associated with CRC clinical stages

**FIGURE 2:**
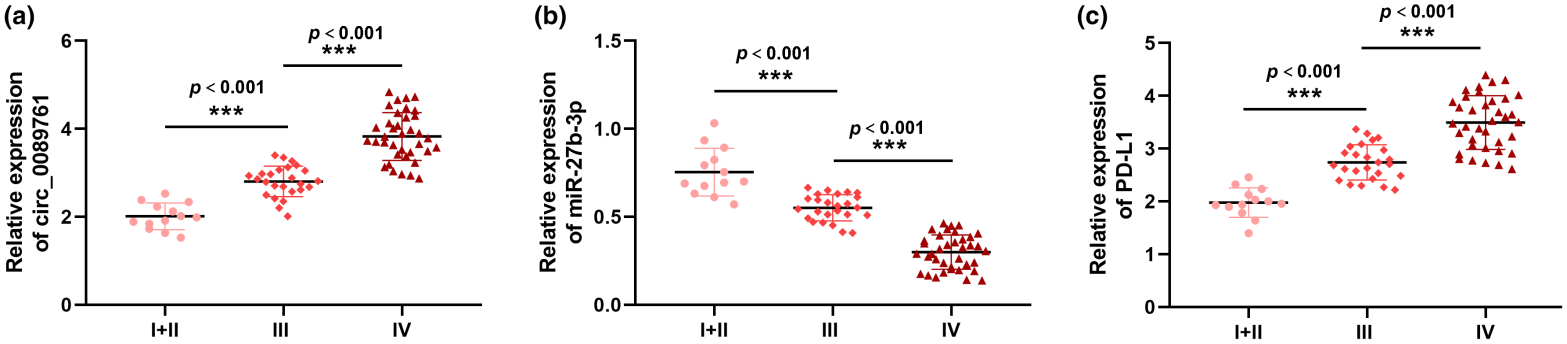
circ_0089761, miR‐27b‐3p and PD‐L1 mRNAs expression in different clinical stages. (a) RT‐qPCR was utilized to quantify circ_0089761 expression in stage I + II, III and IV. (b) RT‐qPCR was utilized to assess miR‐27b‐3p expression in stage I + II, III and IV. (c) RT‐qPCR was utilized to determine PD‐L1 mRNA expression in stage I + II, III and IV. ***p < 0.001 versus I + II stage or III stage.

Generally, the survival rate of cancer patients was directly related to the clinical stages. To evaluate the association between these genes (circ_0089761, miR‐27b‐3p, and PD‐L1) and the clinical stages in CRC, we categorized the clinical stages based on pathological analysis. The clinical stages were classified into three groups: stage I + II (*n* = 13), stage III (*n* = 25), and stage IV (*n* = 37). RT‐qPCR was utilized to measure circ_0089761, miR‐27b‐3p, and PD‐L1 expression CRC tumor samples in different stages. Our results revealed a remarkable association between the clinical stages and the expression levels of these genes. Interestingly, it was observed that circ_008976 expression (Figure 2a) and PD‐L1 (Figure 2c) exhibited a gradual increase as the clinical stages advanced (*p* < 0.001). Conversely, miR‐27b‐3p (Figure 2b) significantly decreased with the progression of clinical stage (*p* < 0.001). The findings provided further support for the hypothesis that the circ_0089761/miR‐27b‐3p/PD‐L1 axis could play critical roles in CRC development.

### 3.3 High correlation was observed among circ_0089761, miR‐27b‐3p, and PD‐L1 in CRC tumor samples

**FIGURE 3:**
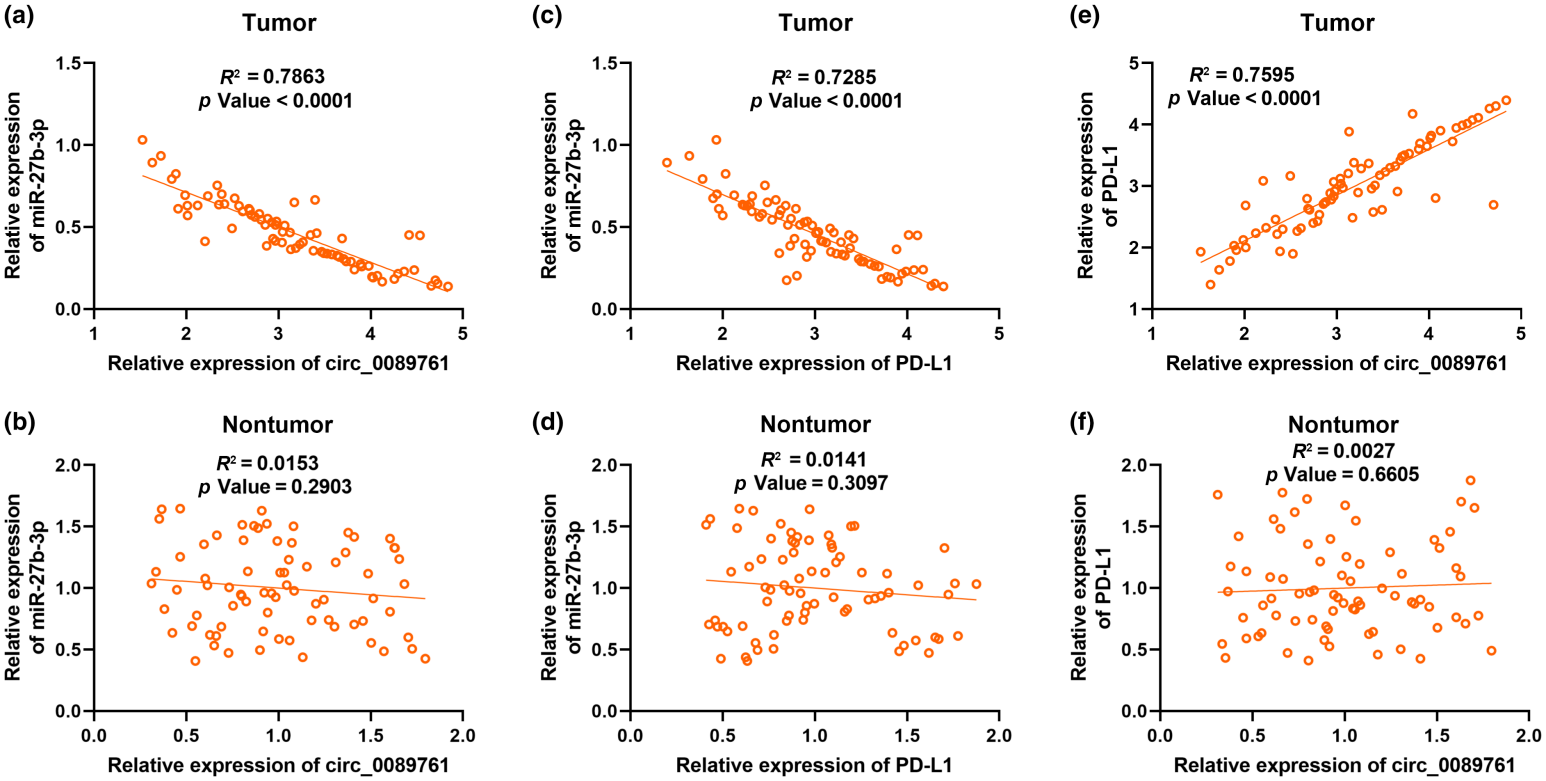
Pearson's correlation assay among circ_0089761, miR‐27b‐3p, and PD‐L1 in CRC tumor and nontumor tissues. Correlations between circ_0089761 and miR‐27b‐3p in CRC (a) and nontumor tissues (b) were analyzed with Pearson's correlation coefficient. Correlations between miR‐27b‐3p and PD‐L1 in CRC (c) and nontumor tissues(d) were analyzed with Pearson's correlation coefficient. Correlations between circ_0089761 and PD‐L1 in CRC (e) and nontumor tissues (f) were analyzed with Pearson's correlation coefficient.

Correlation analysis commonly indicates potential interactions in cancer research. Pearson's correlation coefficient was used to assess the correlation among circ_0089761, miR‐27b‐3p, and PD‐L1. The analysis revealed significant correlations among these three factors in tumor samples. Notably, circ_0089761 was negatively associated with miR‐27b‐3p in tumor (Figure 3a). Similarly, miR‐27b‐3p also exhibited a negative association with PD‐L1 (Figure 3c), whereas circ_0089761 showed a positive correlation with PD‐L1 in tumor (Figure 3e). However, there was no significant correlation observed among circ_0089761, miR‐27b‐3p, and PD‐L1 in nontumor samples (Figure 3b,d,f). These findings provide valuable insights into the interplay of circ_0089761, miR‐27b‐3p, and PD‐L1 in the context of cancer development.

### 3.4 circ_0089761 increased PD‐L1 in CRC cells via targeting miR‐27b‐3p

**FIGURE 4:**
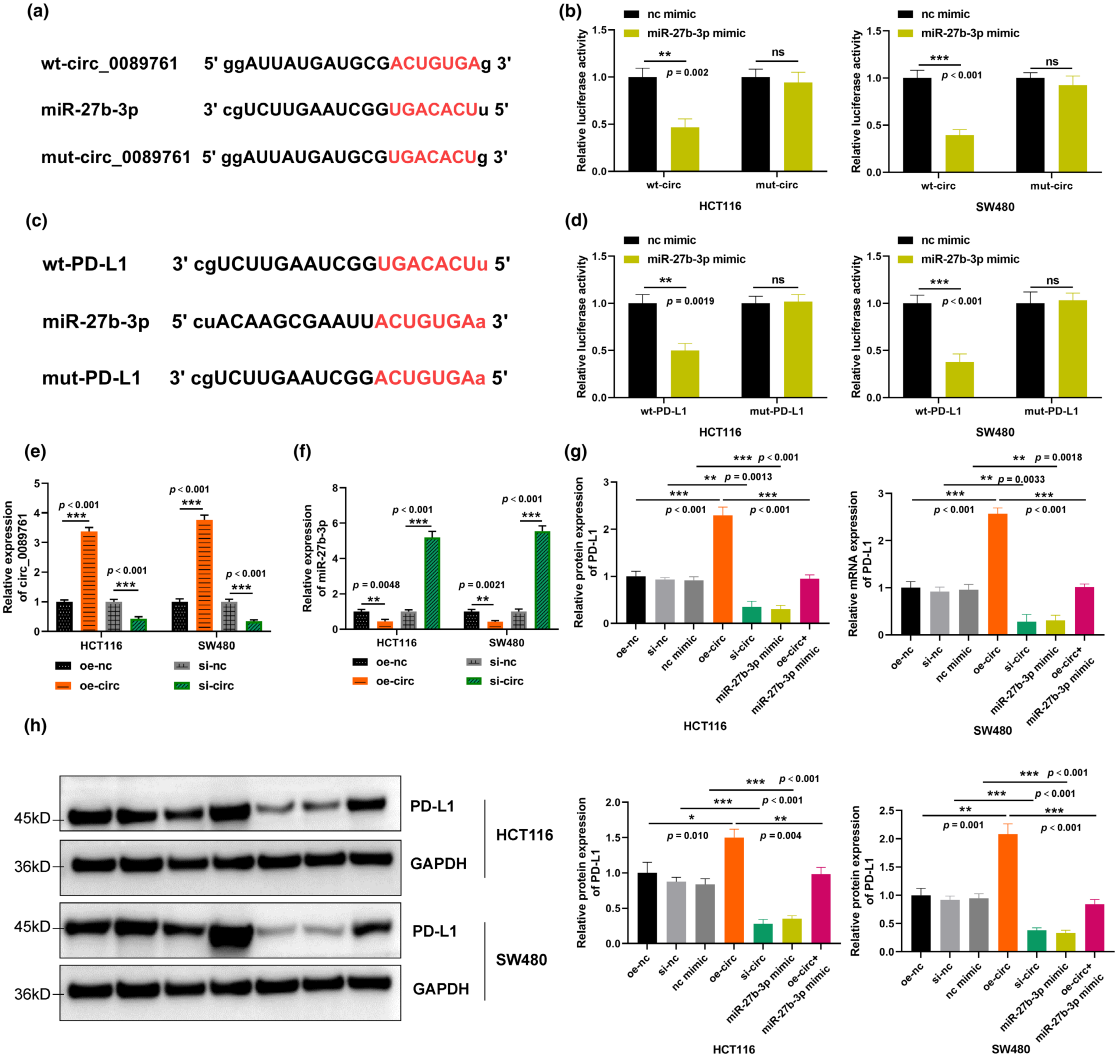
circ_0089761 regulated PD‐L1 in CRC cells via targeting miR‐27b‐3p. (a) The potential binding sequence between circ_0089761 and miR‐27b‐3p was predicted by starbase online software. (b) The binding relationship between circ_0089761 and miR‐27b‐3p was verified by luciferase reporter assay. (c) The potential binding sequence between miR‐27b‐3p and PD‐L1 was predicted by starbase online software. (d) The binding relationship between miR‐27b‐3p and PD‐L1 was validated by luciferase reporter assay. (e) RT‐qPCR was utilized to examine the efficiency of circ_0089761 overexpression and knockdown in HCT116 and SW480 cells. (f) RT‐qPCR was used to measure miR‐27b‐3p expression after circ_0089761 overexpression and knockdown in HCT116 and SW480 cells. (g) RT‐qPCR was utilized to measure PD‐L1 mRNA expression in HCT116 and SW480 cells after different transfection. (h) Western blotting was conducted to measure PD‐L1 protein expression in HCT116 and SW480 cells after different transfection. *p < 0.05 versus oe‐nc; **p < 0.01 versus nc mimic, oe‐nc, si‐nc, or oe‐circ; ***p < 0.001 versus nc mimic, oe‐nc, si‐nc, or oe‐circ.

RNA‐mediated interactions have been implicated in various functional processes. To further demonstrate the potential functions of circ_0089761/miR‐27b‐3p/PD‐L1 axis in CRC, starbase online software was utilized to predict the potential binding sequence and luciferase reporter assay was conducted to confirm their bindings. Firstly, the potential binding sequence of circ_0089761 and miR‐27b‐3p was predicted, as illustrated in Figure 4a. Subsequent luciferase reporter results indicated that circ_0089761 directly bound to miR‐27b‐3p (Figure 4b). Similar experiments were conducted to demonstrate that miR‐27b‐3p directly bound to PD‐L1 (Figure 4c,d).

To explore the roles of circ_0089761 in CRC, silencing circ_0089761 and circ_0089761 overexpression cells (HCT116 and SW480 cell lines) were employed to conduct the function experiments. Initially, the circ_0089761 expression in these cells was measured. As anticipated, knockdown of circ_0089761 significantly decreased the circ_0089761 expression, while circ_0089761 overexpression (oe‐circ_0089761) remarkably increased the circ_0089761 expression in HCT116 and SW480 cell lines (Figure 4e). Moreover, oe‐circ_0089761 significantly inhibited the miR‐27b‐3p expression, whereas circ_0089761 knockdown remarkably promoted miR‐27b‐3p expression (Figure 4f). PD‐L1 expression was significantly enhanced by circ_0089761 overexpression, while it was suppressed by circ_0089761 silencing and miR‐27b‐3p mimic. However, miR‐27b‐3p mimic could reverse the functions of oe‐circ_0089761 on PD‐L1 in CRC cell lines (Figure 4g). Additionally, Western blotting was employed to measure the relative protein expression to study the effect of circ_0089761 on PD‐L1 protein expression. The results indicated that oe‐circ_0089761 significantly stimulated the PD‐L1 protein expression, while silencing circ_0089761 remarkably suppressed the PD‐L1 expression. Similarly, miR‐27b‐3p mimic also reversed the functions of oe‐circ_0089761 on PD‐L1 protein expression in HCT116 and SW480 cells (Figure 4h). In sum, these findings suggested that circ_0089761 upregulated PD‐L1 through inhibiting miR‐27b‐3p, which accelerated CRC progression.

### 3.5 Circ_0089761 knockdown inhibited CRC progression via targeting miR‐27b‐3p/PD‐L1 axis

**FIGURE 5:**
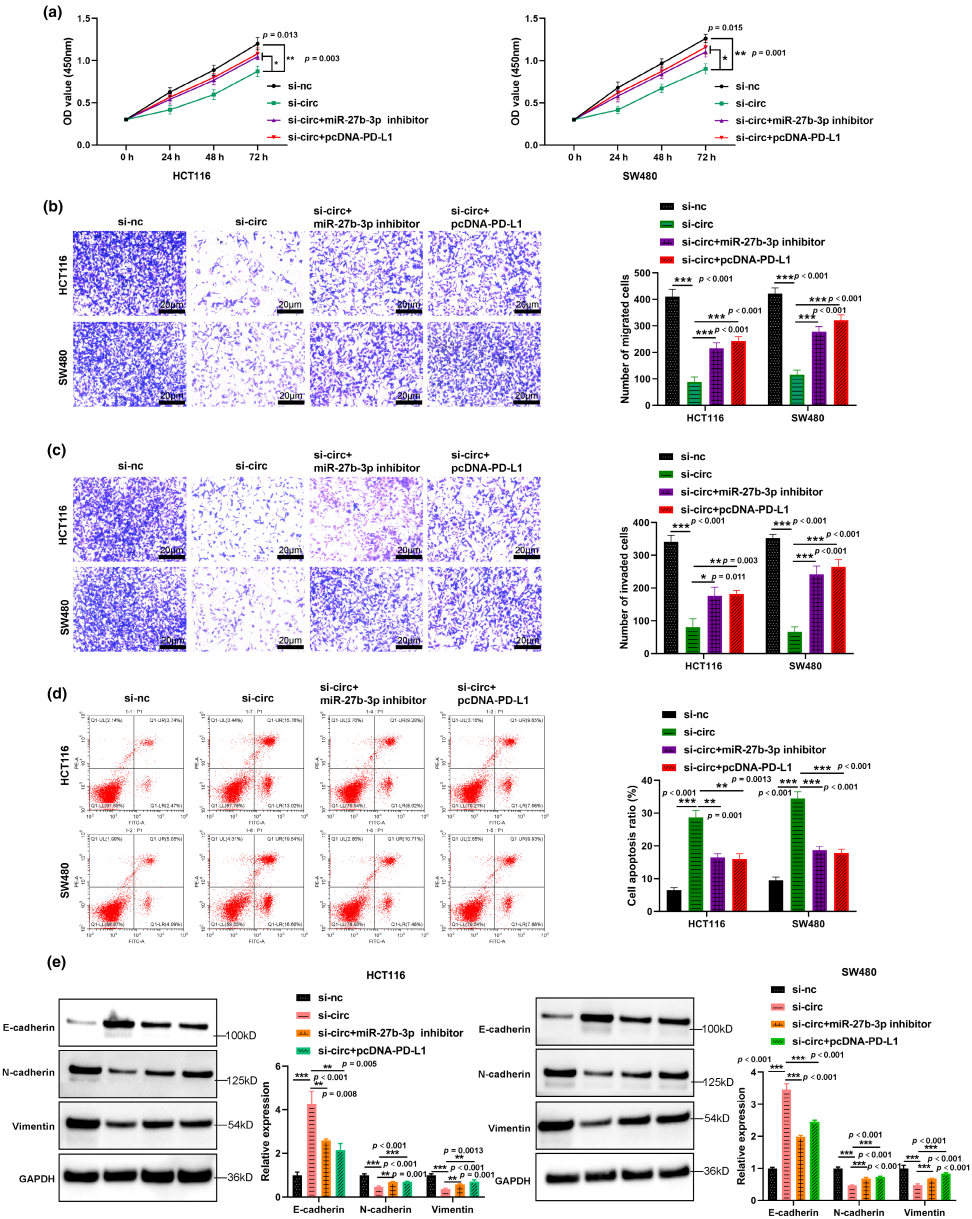
circ_0089761 knockdown inhibited CRC progression via targeting miR‐27b‐3p/PD‐L1 axis. (a) CCK‐8 assay was utilized to detect cell proliferation in HCT116 and SW480 cells after different transfection. Transwell assay was utilized to detected cell migration (b) and invasion (c) ability in CRC cells after different transfection. Scale bars: 20 μm. (d) Flow cytometry was used to measure cell apoptotic rate. (e) Detection of E‐cadherin, N‐cadherin and vimentin expression in CRC cells using western blotting. *p < 0.05 versus si‐circ; **p < 0.01 versus si‐nc or si‐circ; ***p < 0.001 versus si‐nc or si‐circ.

To further explore the potential functions of circ_0089763/miR‐27b‐3p/PD‐L1 axis in CRC progression, various cellular processes were evaluated, including cell proliferation, migration, invasion ability, and apoptotic rate. We utilized si‐circ_0089763 to knock down circ_0089763 expression and observed a significant decrease in cell proliferation (Figure 5a), migration (Figure 5b), and invasion (Figure 5c) in HCT116 and SW480 cells. Conversely, circ_0089763 knockdown significantly increased cell apoptotic rate. Notably, both knockdown of miR‐27b‐3p and overexpression of PD‐L1 could reverse the effect of silencing circ_0089763 on cell proliferation, migration, invasion, and apoptotic rate (Figure 5a–d). Furthermore, we observed that knockdown of circ_0089761 resulted in low expression of mesenchymal markers (N‐cadherin, vimentin) and increased expression of epithelial markers (E‐cadherin), whereas inhibition of miR‐27b‐3p or overexpression of PD‐L1 had the opposite effect (Figure 5e). These results demonstrated the involvement of the circ_0089761/miR‐27b‐3p/PD‐L1 axis in regulating CRC progression and emphasized the potential therapeutic significance of targeting this axis in CRC progression.

### 3.6 Loss of circ_0089761 attenuated CRC tumor growth in vivo

**FIGURE 6:**
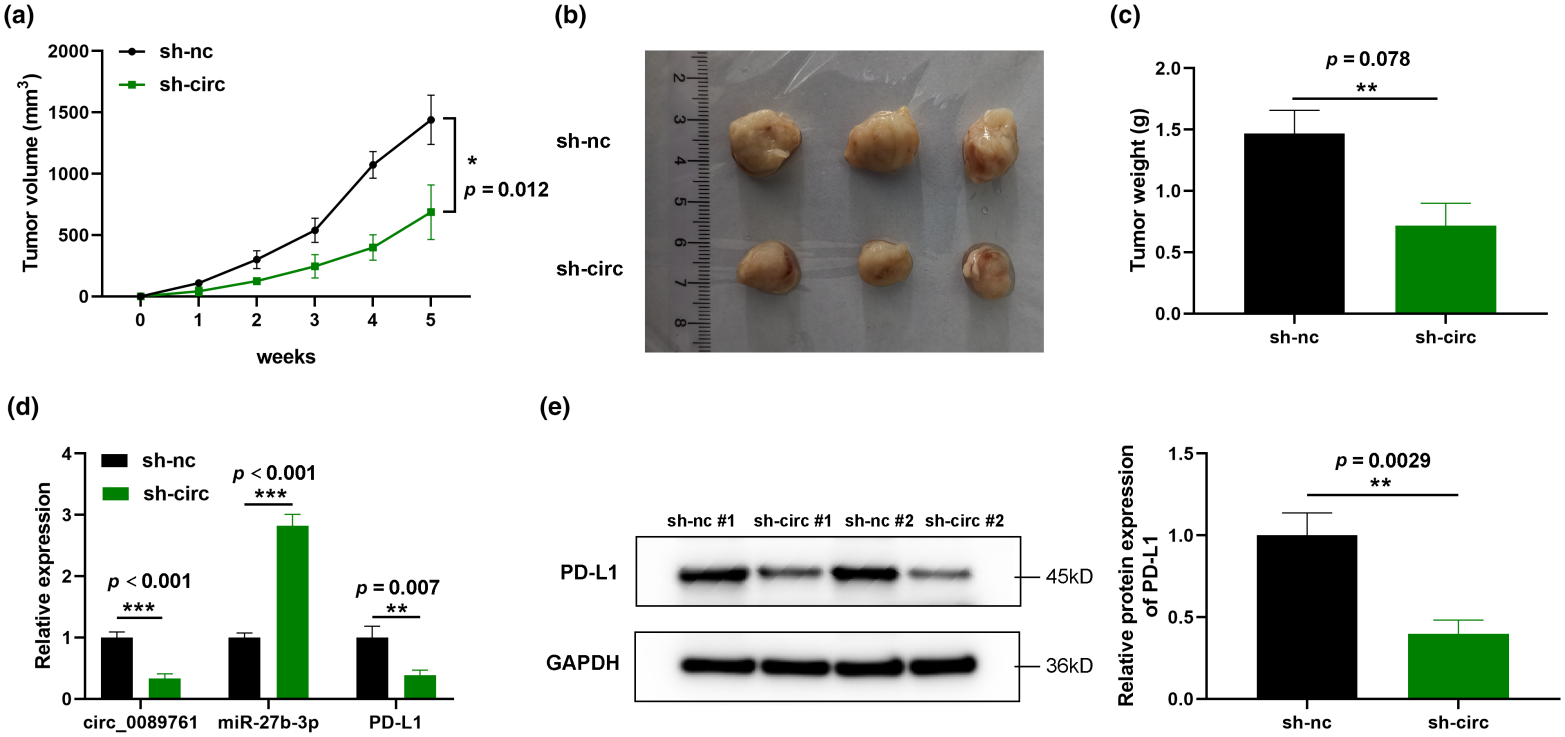
Silencing circ_0089761 attenuated CRC tumor size in vivo. (a) Tumor volume was measured weekly. (b) Tumor images of sh‐NC or sh‐circ_0089761 group in 5th week. (c) Tumor weight of sh‐NC or sh‐circ_0089761 group was measured in 5th week. (d) Expression of circ_0089761, miR‐27b‐3p, and PD‐L1 in tumor samples was determined by RT‐qPCR. (e) Western blotting was utilized to measure the PD‐L1 protein expression in tumor samples. *p < 0.05 versus sh‐nc; **p < 0.01 versus sh‐nc; ***p < 0.001 versus sh‐nc.

To evaluate the functions of circ_0089761 on the development of CRC in vivo, mice model was established by intravenously injecting CT26 cells, which stably expressed either control or sh‐circ_0089761. Tumor volume was assessed weekly (volume = (length×width^2^)/2). The results demonstrates that sh‐circ_0089761 significantly decreased in both tumor volume (Figure 6a,b) and weight (Figure 6c). Additionally, to verify the functions of circ_0089761/miR‐27b‐3p/PD‐L1 axis in vivo, RT‐qPCR and Western blot were utilized to measure their mRNA and protein expression after sh‐circ_0089761 transfection. The results showed that sh‐circ_0089761 significantly inhibited the circ_008976 and PD‐L1 expression, while miR‐27b‐3p was significantly increased (Figure 6d). Additionally, sh‐circ_0089761 significantly suppressed the PD‐L1 protein expression (Figure 6e). This animal experiments provided further support for the hypothesis that the circ_0089761/miR‐27b‐3p/PD‐L1 axis may play critical functions in CRC development.

### 3.7 Silencing circ_008976 impeded antitumor CD8 + T cell depletion

**FIGURE 7:**
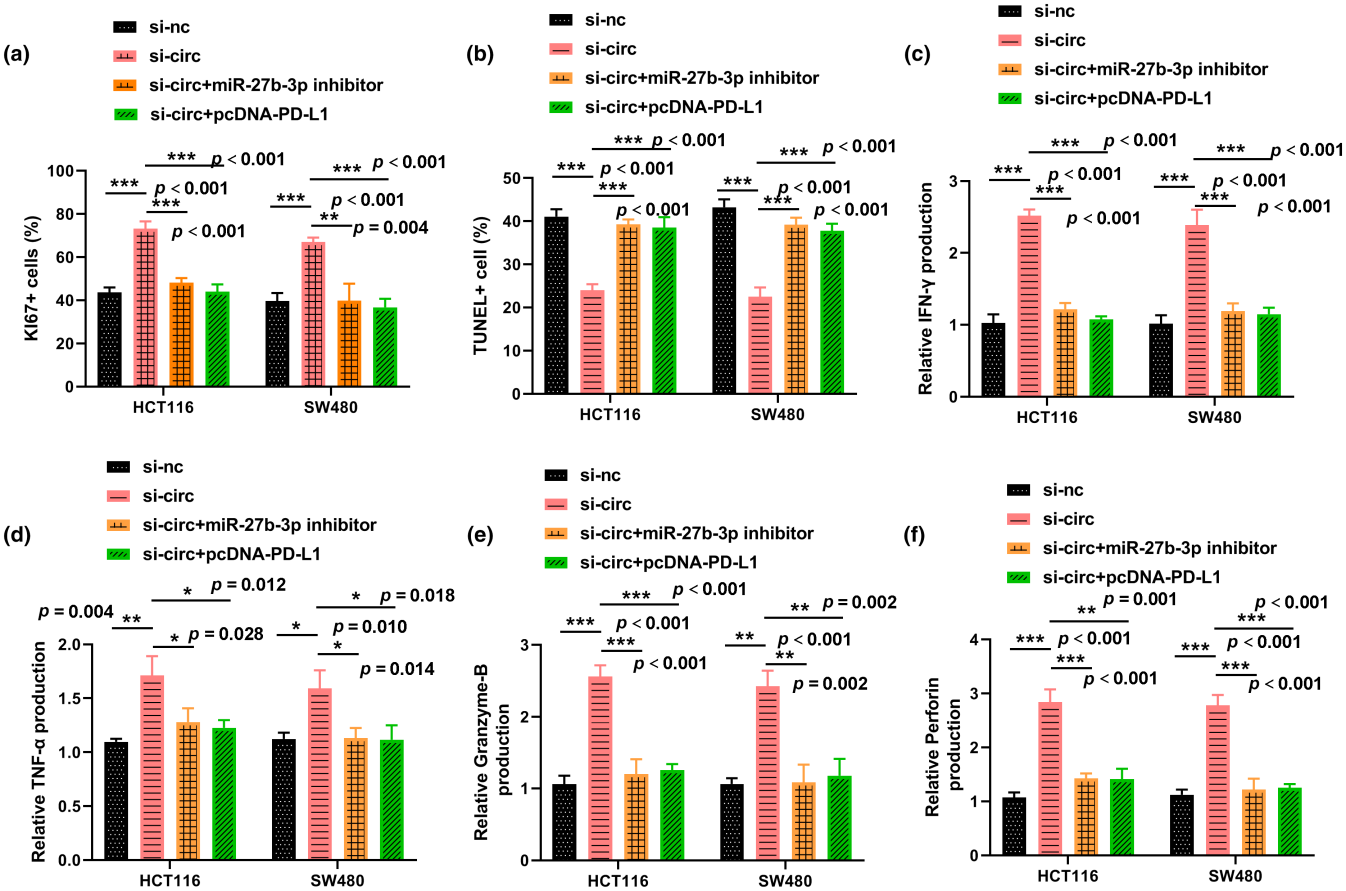
Silencing circ_0089761 facilitated the proliferation of CD8+ T cells. (a) The proliferative capacity of CD8 + T cells was assessed by measuring Ki67 expression in CD8 + T cells by flow cytometry. (b) Apoptosis of CD8+ T cells was evaluated by TUNEL assay. (c–f) ELISA was applied to detect the levels of IFN‐γ, TNF‐α, perforin, and granzyme‐B in CD8+ T cell supernatants. *p < 0.05 versus si‐nc or si‐circ; **p < 0.01 versus si‐nc or si‐circ; ***p < 0.001 versus si‐nc or si‐circ.

Infiltrating CD8 + T cells are recognized as the most important antitumor cell type; therefore, we investigated the effect of the circ_0089761/miR‐27b‐3p/PD‐L1 axis on CD8 + T cells. To prevent the direct contact between cells, we cocultured CRC cells with isolated CD8 + T cells using the Transwell coculture system. The results showed that silencing circ_0089761 in CRC cells significantly exacerbated CD8 + T cell proliferation (Figure 7a) and inhibited cell death (Figure 7b) in the coculture system. In contrast, knockdown of miR‐27b‐3p or overexpression of PD‐L1 in CRC cells partially impeded this effect, suggesting that circ_0089761/miR‐27b‐3p/PD‐L1 axis in CRC cells was involved in CD8 + T cell growth and death. Furthermore, ablation of circ_0089761 in CRC cells also increased the secretion of IFN‐γ, TNF‐α, perforin, and granzyme‐B in CD8 + T cells (Figure 7c–f), whereas the increase in cytokine secretion induced by si‐circ_0089761 was partially suppressed after miR‐27b‐3p depletion or PD‐L1 overexpression. These results suggested that targeting the circ_0089761/miR‐27b‐3p/PD‐L1 axis might enhance CD8 + T function.

## 4 DISCUSSION

In general, biomarkers, such as lncRNA (Gao et al., 2021; Wu et al., 2022), circRNA (Zhang & Dai, 2022; Zhou et al., 2020), miRNA (Diener et al., 2022; Fabian & Sonenberg, 2012), and protein (Liu, Zhou, et al., 2021; Ryu et al., 2021), are of significant importance in cancer treatment. The circRNA‐miRNA‐protein axis has potential for tumor therapy (Chen et al., 2020; Li et al., 2020; Martín et al., 2021). In this study, we identified a relationship among circ_0089761 and some clinical characteristics in CRC, such as tumor size and TNM staging. Notably, circ_0089761 and PD‐L1 was significantly increased in CRC tissue, while miR‐27b‐3p was remarkably decreased. Bioinformatics analysis indicates that circ_0089761/ miR‐27b‐3p/PD‐L1 axis might have crucial functions in CRC development. In brief, circ_0089761 was upregulated in CRC and promoted PD‐L1 expression through inhibiting miR‐27b‐3p.

The utilization of circRNAs as diagnostic biomarkers holds great potential in cancer research. Numerous studies have confirmed that circRNAs, owing to their abundance and stability during development, can serve as diagnosis biomarkers in some cancers (Chen et al., 2021; Li et al., 2020; Yu et al., 2022; Zhang et al., 2019). For instance, the circNRIP1 transcript has been identified to be a prominent biomarker for gastric cancer (Zhang et al., 2019), while circ_0003258 has been confirmed as a biomarker for prostate cancer (Yu et al., 2022). Additionally, circUSP7 has exhibited biomarker properties in the context of lung cancer (Chen et al., 2021). Circ_0089761 exhibits a strong capability as a miRNA sponge owing to its remarkable sequence length and its localized presence in the cytoplasm (Liu et al., 2022). Our findings indicated a significant association between circ_0089761 and tumor size as well as TNM staging in tumor. Moreover, circ_008976 expression markedly increase with the progression of clinical stages. However, there is a paucity of research on circ_0089761 so far and its underlying mechanisms remain elusive.

Emerging research suggested that circRNAs mediated their biological functions through the interaction with miRNAs, which in turn regulate the protein expression. For instance, CircRAB11FIP1 promotes ovarian cancer via miR‐129/DSC1 axis (Zhang, Zhu, & Hu, 2021), circEPSTI1 upregulates SLC7A11 expression in cervical cancer by miR‐375 (Wu et al., 2021) and circ_0110389 accelerates gastric cancer through miR‐127‐5p/SORT1 (Liang et al., 2021). The aberrant expression of PD‐L1 was observed in numerous cancers, including CRC (Payandeh et al., 2020), cervical cancer (Liang et al., 2022), and lung cancer (Koh et al., 2021). In this study, the starbase online software was performed to predict the miRNAs which bound to circ_0089761, and the miRNA's binding proteins. The results found that circ_0089761 was potentially bound to miR‐27b‐3p, which was bound to PD‐L1. Then, luciferase reporter assays were performed to confirm the direct binding relationship. Pearson's correlation analysis revealed a significant correlation among circ_0089761, miR‐27b‐3p, and PD‐L1 in CRC in tumor. Thus, it was hypothesized that circ_0089761/miR‐27b‐3p/PD‐L1 axis could play crucial functions in CRC.

Given the heterogeneous nature of tumors, miR‐27b‐3p has been identified as having dual functions, acting as either an oncogenic or tumor‐suppressive factor in the context of tumor progression (Sun et al., 2020). For example, miRNA‐27b promotes breast cancer via PDHX suppression (Eastlack et al., 2018). Conversely, miR‐27b‐3p downregulates LIMK1 expression, inhibiting CRC development (Chen et al., 2017). PD‐L1 plays a critical role in immune regulation through its interaction with PD‐1 in immune cells (Topalian et al., 2020). To unravel the mechanistic intricacies underlying the circ_0089761/miR‐27b‐3p/PD‐L1 axis in CRC, a comprehensive investigation was conducted to dissect its regulatory pathways. We found that silencing circ_0089761 increased the miR‐27b‐3p and decreased PD‐L1. On the contrary, oe‐circ_0089761 suppressed the miR‐27b‐3p and stimulated PD‐L1. Notably, miR‐27b‐3p mimic was observed to counteract the effects of circ_0089761 on PD‐L1. These results were supported by Pearson's correlation assay. Therefore, it was hypothesized that circ_0089761 upregulated PD‐L1 through inhibiting miR‐27b‐3p, which accelerated CRC progression.

CD8+ T cell dysfunction‐mediated impairment of antitumor immunity is an important feature of cancer (Thommen & Schumacher, 2018). In addition, circRNAs have been implicated in tumor immune evasion. For example, the non‐small cell lung cancer cell‐derived exosomal circUSP7 promoted CD8 + T cell exhaustion (Chen et al., 2021). The exosomal circTRPS1 secreted by bladder cancer cells induced CD8 + T cell failure (Yang et al., 2022). In this study, we utilized a CD8 + T and CRC cell coculture system to investigate whether circ_0089761/miR‐27b‐3p/PD‐L1 was involved in the formation of the immunosuppressive microenvironment of CRC. Our data showed that CD8 + T cells cocultured with si‐circ_0089761‐transfected CRC cells exhibited enhanced proliferative capacity and produced higher amounts of IFN‐γ, TNF‐α, perforin, and granzyme‐B. Conversely, miR‐27b‐3p depletion or PD‐L1 overexpression reduced si‐circ_0089761 induced tumor killing ability of CD8 + T cells. Thus, circ_0089761 might be a promising target for enhancing antitumor immunotherapy.

Our study also has its limitations, which are worth mentioning. Although our findings might provide a novel mechanism to explain the link between high circRNA expression in CRC and high PD‐L1 expression. However, whether circ_0089761 regulates PD‐L1 levels through other mechanisms remains elusive, and we will focus on this question in our future work.

## 5 CONCLUSIONS

Overall, this study identified that circ_0089761, miR‐27b‐3p, and PD‐L1 were intimately related to CRC formation and progression, which could be a therapeutic target for CRC. The circ_0089761/miR‐27b‐3p/PD‐L1 axis plays vital roles in cell proliferation, invasion, migration, and apoptotic rate. Mechanistically, circ_0089761 upregulated PD‐L1 through inhibiting miR‐27b‐3p. Therefore, circ_0089761/miR‐27b‐3p/PD‐L1 axis could holds promising prospects as therapeutic strategy for CRC.

## AUTHOR CONTRIBUTIONS

Qizhong Gao and Xiaowei Cheng:were involved in conceptualization, methodology, and writing‐original draft. Xiang Gao was involved in supervision, writing‐review and editing. All authors read and approved the final version of the manuscript.

## FUNDING INFORMATION

This research was supported by Wuxi Municipal Health Commission on the Project (M202215), and Research Project of Cutting‐Edge Tumor Support Therapy (nphcf‐2022‐118).

## CONFLICT OF INTEREST STATEMENT

The authors declare that they have no conflicts of interest to report regarding the present study.

## ETHICS STATEMENT

This study was approved by the Ethics Committee of Affiliated Hospital of Jiangnan University. All participants were provided with written informed consent at the time of recruitment, and all experiments involving human tissue specimens comply with the Declaration of Helsinki. Animal studies were performed in compliance with the ARRIVE guidelines.

## Data Availability

The data used to support the findings of this study are available from the corresponding author upon request.
